# Intracranial infection in an adult caused by *Mycoplasma hominis*, diagnosed using mNGS technology: a case report

**DOI:** 10.3389/fmed.2025.1560635

**Published:** 2025-03-04

**Authors:** Kai-Meng Wang, Na Mu, He-Bo Wang

**Affiliations:** ^1^The Neurology Department of Hebei Provincial People's Hospital, Shijiazhuang, Hebei, China; ^2^Department of Clinical Laboratory of Examination, Harrison International Peace Hospital, Hengshui, Hebei, China

**Keywords:** *Mycoplasma hominis*, adults, meningoencephalitis, metagenomic next-generation sequencing, diagnosis, case report

## Abstract

*Mycoplasma hominis* is a rare cause of adult central nervous system infections, posing significant diagnostic challenges due to its fastidious growth requirements and high false-negative rate in conventional cultures. We report a case of *Mycoplasma hominis* meningoencephalitis in a postpartum female, diagnosed via metagenomic next-generation sequencing (mNGS) of cerebrospinal fluid (CSF). The patient presented with fever, headache, and progressive neurological deficits following a cesarean section. Neuroimaging revealed a subdural hematoma, and CSF analysis demonstrated an inflammatory response. mNGS identified *Mycoplasma hominis*, prompting targeted antimicrobial therapy with moxifloxacin and doxycycline, which led to significant clinical improvement. This case underscores the utility of mNGS in detecting rare intracranial infections and highlights the critical role of early pathogen identification in optimizing treatment outcomes.

## Introduction

*Mycoplasma hominis* is a member of the Mycoplasma family, commonly found as a commensal in the human oral cavity, respiratory tract, and urogenital tract, and is a frequent pathogen in neonatal infections (1). *Mycoplasma hominis* is associated with pelvic inflammatory disease (PID) and can contribute to pregnancy-related and postpartum complications, including preterm birth, chorioamnionitis, and postpartum endometritis. Its role in these conditions is linked to its ability to colonize the female genital tract and trigger inflammatory responses (2). *Mycoplasma hominis* is capable of symbiotically residing and replicating within *Trichomonas vaginalis*. Research has shown that *M. hominis* can persist in the cytoplasm of *T. vaginalis*, utilizing the host’s metabolites for growth. This symbiotic relationship may provide M. hominins with a protective niche against the host immune response while potentially modulating the pathogenicity of *T. vaginalis* (3).

While *Mycoplasma hominis* is often associated with infections of the external genitalia in adults, postoperative wound infections, and septic arthritis, its involvement in central nervous system infections is relatively rare (1, 4). Due to the absence of a cell wall, *Mycoplasma hominis* is challenging to grow in conventional *in vitro* cultures. It has long detection cycles and a high rate of false positives (5).

Metagenomic next-generation sequencing (mNGS) is an effective method for pathogen detection and can be used to identify Mycoplasma (6, 7). This study reports a case of *Mycoplasma hominis* meningoencephalitis diagnosed through cerebrospinal fluid (CSF) mNGS in a patient after cesarean section. This case contributes to a better understanding of the clinical characteristics of Mycoplasma meningoencephalitis, helping reduce misdiagnosis and missed diagnoses by clinicians.

## Case report

**Figure 1:**
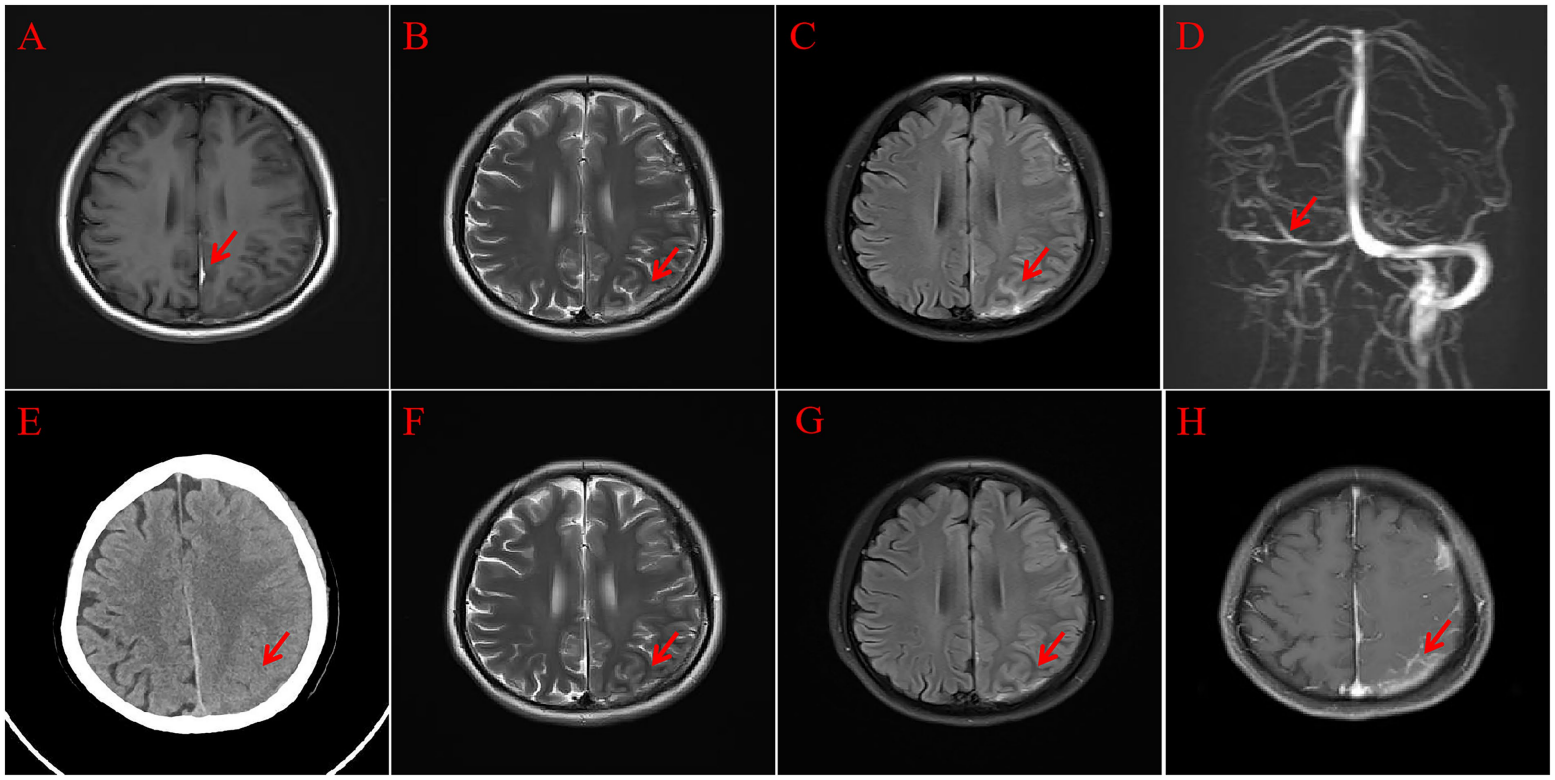
Head MRI. T1 **(A)** Shows a subdural hematoma (red arrow); T2 **(B)** shows cortical swelling in the left cerebral hemisphere (red arrow); Flair **(C)** shows the subdural hematoma rupturing into the subarachnoid space (red arrow); MRV **(D)** shows abnormal development of the right transverse and sigmoid sinuses (red arrow); follow-up head CT **(E)** shows improvement in the left cortical swelling (red arrow); follow-up head MRI, T2 **(F)**, and Flair **(G)** show reduced left cortical swelling (red arrow); enhanced MRI **(H)** shows abnormal enhancement in the subdural area and some sulci in the left frontal-temporal–parietal region (red arrow).

**Figure 2:**
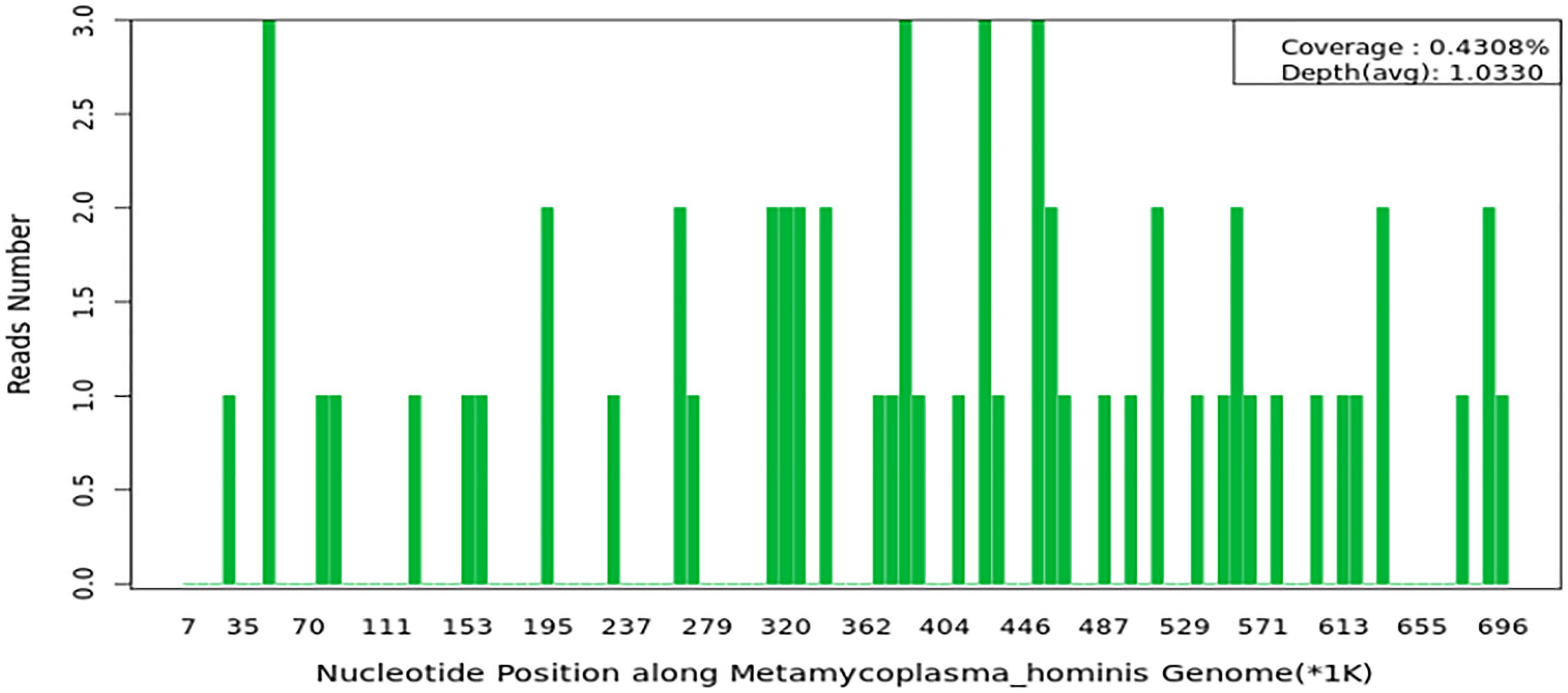
The cerebrospinal fluid mNGS results.

A female patient in her 30s was admitted to our hospital due to persistent headaches and fever for 10 days, along with weakness in the right limbs for 3 days. The patient had a history of cesarean section 13 days prior and denied any history of trauma. Physical examination revealed a temperature of 38.2°C, a heart rate of 104 beats and a respiratory rate of 20 breaths per minute, and blood pressure of 135/85 mmHg. Muscle strength in the right limbs was 4+, with reduced pain and temperature sensation on the right side and positive neck stiffness. Blood tests showed a white blood cell (WBC) count of 12.19 × 10^9^/L and a neutrophil count of 10.87 × 10^9^/L, with normal coagulation and biochemical results. After the physical examination, the patient was preliminarily diagnosed with a high likelihood of intracranial infection. Given that she is a postpartum female presenting with headache and focal neurological deficits, further head Magnetic Resonance Imaging (MRI) examination was recommended to assist in differentiating intracranial venous sinus thrombosis. Additionally, to rule out the possibility of stroke, a head MRI with Diffusion Weighted Imaging (DWI) was performed. We also recommended additional diagnostic procedures, including lumbar puncture, head MRI, and pathogen testing. Head MRI and Magnetic Resonance Venography (MRV) indicated a left frontal-temporal–parietal subdural hematoma ruptured into the subarachnoid space, and MRV revealed abnormal development of the right transverse and sigmoid sinuses (Figure 1). On the second day of admission, the patient’s right limb weakness worsened, accompanied by persistent high fever. Examination revealed right-sided facial weakness with a shallow nasolabial fold and tongue deviation, grade 2 muscle strength in the right limbs, positive Babinski sign, reduced pain and temperature sensation, and neck stiffness. Head CT showed unclear gray-white matter boundaries and brain swelling in the left cerebral hemisphere (Figure 1). On the third day, a lumbar puncture revealed a pressure of 240 mmH2O. CSF analysis showed a total protein concentration of 706 mg/L and a WBC count of 197 × 10^6^. The mNGS of CSF identified *Mycoplasma hominis* with 148 sequences detected (Figure 2). The patient was diagnosed with *Mycoplasma hominis* meningoencephalitis.

**Figure 3:**
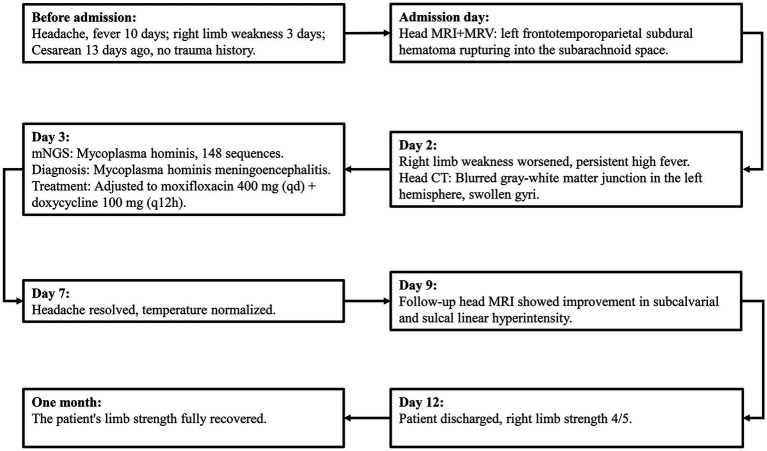
The detailed diagnosis and treatment process of the patient.

The treatment strategy was adjusted to moxifloxacin 400 mg (intravenous injection) once daily and doxycycline 100 mg (oral administration) every 12 h. The patient’s headache resolved one week after admission, and body temperature returned to normal. MRI on the 11th day showed improved left frontal-temporal–parietal subdural and sulcal hyperintensity. Abnormal enhancement was observed in the left frontal-temporal–parietal subdural area and some sulci (Figure 1). A lumbar puncture on the 12th day showed a pressure of 120 mmH2O, with no significant abnormalities in CSF routine and biochemical tests. The patient was discharged on the 14th day after improvement. Upon discharge, a slightly shallow right nasolabial fold and slight right tongue deviation were noted; muscle strength in the right limbs was grade 4, and the right Babinski sign remained positive. One month post-discharge, follow-up showed the patient’s limb strength had fully recovered. The patient’s detailed diagnosis and treatment process is summarized in Figure 3.

## Discussion

*Mycoplasma hominis* is a pathogenic species of Mycoplasma in humans, commonly causing urogenital infections such as urethritis, cervicitis, pelvic inflammatory disease, pregnancy complications, and neonatal infections (8, 9). With advances in diagnostic technology, there have been reports of Mycoplasma causing central nervous system changes in children, including encephalitis, optic neuritis, acute psychiatric symptoms, stroke, cranial nerve palsies, aseptic meningitis, Acute Disseminated Encephalomyelitis (ADEM), Guillain-Barré Syndrome (GBS), and transverse myelitis (10). However, reports of intracranial infections caused by *Mycoplasma hominis* in adults remain relatively rare.

In this study, the patient presented with fever and headache accompanied by positive meningeal signs. Lumbar puncture CSF analysis indicated an inflammatory response, which is not significantly different from the clinical features of intracranial infections caused by other pathogens. Thus, diagnosing intracranial Mycoplasma infections based solely on clinical symptoms and laboratory tests is challenging. Additionally, detecting Mycoplasma is difficult, time-consuming, and has a high false-negative rate (5).

mNGS is a novel genetic testing method that performs high-throughput sequencing of nucleic acids directly from clinical samples, comparing them with databases to identify the types of pathogens present in the sample. It can quickly detect specific pathogens. Study have shown that mNGS plays an important auxiliary role in diagnosing unexplained encephalitis (11). mNGS enables the unbiased detection of nucleic acids from various pathogens in clinical specimens, including bacteria, fungi, viruses, and parasites. In 2014, the first reports emerged on the use of mNGS for diagnosing central nervous system (CNS) infections (12). Since 2015, CSF mNGS has been increasingly utilized in China for the diagnosis of CNS infectious diseases (13). Subsequent studies have reported that the detection rate of pathogenic organisms in CSF using mNGS ranges from 15.7 to 57.0% (11, 14). Among CNS infection cases, the concordance rate between mNGS and conventional pathogen detection methods ranges from 22.5 to 52.6%. The sensitivity and specificity of CSF mNGS in diagnosing encephalitis and meningitis have been reported as 73 and 99%, respectively (15). These findings underscore the practical value of CSF mNGS in the etiological diagnosis of CNS infectious diseases. Although mNGS technology is associated with high medical expenses, we strictly regulate its clinical indications, recommending CSF mNGS only for patients with unknown etiology, poor response to empirical treatment, severe disease, or immunodeficiency (or immunosuppression) presenting with encephalitis, meningitis, or brain abscess. Additionally, technological advancements have led to a gradual reduction in mNGS costs. Through continuous technological refinement, clinical application research, and accumulated experience, CSF mNGS has now become a crucial and practical tool for the differential diagnosis of CNS infections.

Currently, a consensus has been reached on the use of mNGS in patients with acute encephalitis and meningitis in clinical practice: (1) For community-acquired acute encephalitis, meningitis, and non-severe cases, conventional CSF microbiological testing is generally recommended as the initial approach. (2) For severe cases, mNGS is recommended for the initial CSF analysis (16). (3) For patients with a high suspicion of infection by emerging or rare pathogens, mNGS should be prioritized in the initial CSF analysis (17). (4) For patients with chronic CNS infections of unknown etiology, mNGS is the preferred method for pathogen detection (16). (5) For patients with primary immunodeficiency, neutropenia, AIDS, or those receiving immunosuppressive therapy, mNGS should be performed during the initial CSF evaluation (18). (6) In cases of strong clinical suspicion of RNA virus infection, mNGS should be the first-line diagnostic method (17). The patient’s condition worsened, and conventional empirical treatment proved ineffective. Traditional diagnostic methods for Mycoplasma infections face significant challenges in identifying the pathogen, as they are time-consuming and associated with a high false-negative rate, making a definitive diagnosis extremely difficult. This patient was ultimately diagnosed with Mycoplasma infection through CSF mNGS. This case demonstrated the diagnostic value of mNGS in intracranial infections caused by *Mycoplasma hominis*. Furthermore, the rapid adjustment of antibiotic therapy based on sequencing results led to a quick improvement in the patient’s clinical symptoms and a good prognosis, further confirming the advantage of mNGS in assisting the rapid adjustment of treatment strategies.

The mechanisms by which Mycoplasma invades the central nervous system mainly include the following hypotheses: (1) direct contamination during trauma or surgery; (2) dissemination and implantation in the brain following bacteremia secondary to urogenital surgical procedures; (3) direct spread of respiratory tract colonizing bacteria to open wounds through artificial airways (19).

Previous reports have shown that adult Mycoplasma encephalitis is often accompanied by intracranial hemorrhage or subarachnoid hemorrhage (SAH), with most patients having a history of head trauma followed by cranial surgery (20, 21). However, after consulting the patient’s family, no history of trauma was reported, and the patient had no vascular malformations or coagulation disorders. Currently, there is a lack of research directly linking subdural hematoma (SDH) and SAH to Mycoplasma infection. In this patient, the pathway for intracranial Mycoplasma infection might have involved Mycoplasma entering the bloodstream during the cesarean section, causing bacteremia. Coupled with the presence of SAH, this ultimately led to the dissemination and implantation of *Mycoplasma hominis* in the cerebral cortex. However, the etiology of this patient’s subdural hematoma and SAH remains unclear. Further research is needed to confirm the possibility of Mycoplasma entering the bloodstream.

Studies on successful treatment of central nervous system infections caused by *Mycoplasma hominis* often recommend using tetracyclines combined with quinolones, with doxycycline, minocycline, and moxifloxacin showing the best efficacy. The treatment dosage is the same as routine regimens, with the duration for meningitis treatment typically 2–3 weeks (1). In this study, we administered moxifloxacin combined with doxycycline for two weeks, rapidly improving symptoms. The CSF WBC count, protein, and glucose levels returned to normal. The patient’s improvement may be related to the good blood–brain barrier permeability and drug activity of quinolones, especially moxifloxacin. This also highlights the importance and urgency of early identification of the pathogen and timely medication adjustment in patients with intracranial Mycoplasma infections.

Due to the extreme rarity of intracranial infections caused by Mycoplasma, clinicians often find it difficult to diagnose without microbiological evidence. Additionally, the lack of specific clinical manifestations makes distinguishing Mycoplasma meningitis from bacterial or viral meningitis challenging. However, CSF mNGS technology can quickly provide etiological evidence of *Mycoplasma hominis* infection. Mycoplasma is resistant to most commonly used first-line antibiotics and requires specialized treatment, making early diagnosis crucial.

Although mNGS has shown great promise in clinical applications, it still has certain limitations. For instance, mNGS is highly prone to contamination from human host DNA and environmental microbes. Thus, strict adherence to established guidelines and protocols is essential at every stage, including sample collection and processing, library preparation, sequencing, bioinformatics analysis, and clinical interpretation. Optimizing workflows, strengthening quality control, and training specialized personnel are crucial strategies to minimize contamination risks. Moreover, the high cost of mNGS remains a significant obstacle to its widespread adoption. In China, this technology has not yet been incorporated into the medical insurance system, making cost reduction difficult in the short term. Furthermore, as a novel molecular diagnostic tool, mNGS still lacks broad patient acceptance. Its relatively long turnaround time also leads many patients to prefer PCR-based methods, which are faster and more affordable. Thus, expanding the clinical adoption of mNGS will require continuous technological advancements, cost-reduction strategies, and greater awareness among healthcare providers and patients.

## Conclusion

Although mNGS currently faces challenges in terms of cost, pricing, and technology in clinical applications, its overall positive detection rate and sensitivity are markedly superior to traditional pathogen detection methods. CSF mNGS has become an essential and practical technique for the etiological identification of CNS infections. Therefore, for patients with encephalitis, meningitis, or brain abscesses of unknown etiology—especially those with poor responses to empirical treatment, severe disease, or immunodeficiency (or immunosuppression)—we recommend prioritizing CSF mNGS testing. In the future, adopting differentiated, standardized testing workflows tailored to the characteristics of various patients and specimens, combined with analysis strategies based on the specific microbial distribution of individual hospitals, may represent the direction for achieving precise etiological diagnosis of CNS infections.

## Data Availability

The original contributions presented in the study are included in the article/supplementary material, further inquiries can be directed to the corresponding author.
